# Understanding healthcare efficiency—an AI-supported narrative review of diverse terminologies used

**DOI:** 10.1186/s12909-025-06983-5

**Published:** 2025-03-20

**Authors:** Lotte A. Bock, Sanne Vaassen, Walther N. K. A. van Mook, Cindy Y. G. Noben

**Affiliations:** 1 Academy of Postgraduate Medical Education, Maastricht University Medical Centre, P.O. Box 5800, Maastricht, AZ 6202 the Netherlands; 2 School of Health Professions Education, Maastricht University, Maastricht, the Netherlands; 3 Department of Pediatrics, Maastricht University Medical Centre, Maastricht, the Netherlands; 4 Department of Intensive Care Medicine, Maastricht University Medical Centre, Maastricht, the Netherlands

**Keywords:** Cost-effectiveness, Healthcare efficiency, High-value care, Low-value care, Value-based healthcare, Overuse

## Abstract

**Background:**

Physicians have become more responsible for pursuing healthcare efficiency. However, contemporary literature uses multiple terminologies to describe healthcare efficiency. To identify which term is best suitable for medical education to equip physicians to contribute to healthcare efficiency delivery in clinical practice, we performed a narrative review to elucidate these terms' meanings, commonalities, and differences.

**Methods:**

The PubMed-database was searched for articles published in 2019–2024 describing healthcare efficiency terminology. Eligible articles conceptually described and applied relevant terminologies for physicians, while empirical studies and practice-specific articles were excluded. The screening was supported by an open-source artificial intelligence tool (ASReview), which prioritizes articles through machine learning. Two reviewers independently screened the resulting articles, resolving disagreements by consensus. Final eligibility was determined through predefined inclusion criteria.

**Results:**

Out of 3,655 articles identified, 26 met the inclusion criteria. Key terminologies: *cost-effectiveness*, *high-value care*, *low-value care*, and *value-based healthcare,* were identified, and explored into more depth*.* ‘Value’ is central in all terms, but our findings reveal that the perspectives herein differ on what constitutes value. Within *cost-effectiveness,* resource allocation to the population’s needs drives decision-making—maximizing value at population-level. Within *value-based healthcare,* patient-centricity guides decision-making—maximizing value at individual patient-level. *High-value* and *low-value care* are somewhat ambiguous, depending solely on cost-effectiveness results or patient preferences to determine whether care is considered as low or high value.

**Conclusions:**

Cost-effectiveness may be too rigid for patient-physician interactions, while value-based healthcare might not ensure sustainable care. As physicians are both stewards of finite societal resources and advocates of individual patients, integrating cost-effectiveness (resource allocation for population needs) and value-based healthcare (individualized care plans) seems necessary. Both terms emphasize delivering high-value care and avoiding low-value care. We suggest that medical education: (1) train (future) physicians to apply healthcare efficiency principles through case-based discussion, (2) use the cost-effectiveness plane to evaluate treatments, (3) deepen knowledge of diagnostic and treatment procedures’ costs within evidence-based guidelines, and (4) enhance communication skills supporting a healthcare efficiency-driven open shared decision-making with patients.

**Supplementary Information:**

The online version contains supplementary material available at 10.1186/s12909-025-06983-5.

## Background

In recent decades, the healthcare sector has witnessed a technological upsurge in diagnostic and treatment possibilities, leading to a multitude of advancements in diagnostics and treatment options. However, experts estimate that approximately 10–30% of healthcare services provided are wasteful, representing care that could be eliminated without adversely affecting patients [1–3]. This so-called ‘inappropriate’ care might not only waste limited resources but also carry the potential to inflict physical, mental, and/or financial harm upon patients. Concerning the imperative to ensure healthcare sectors’ sustainability, contemporary healthcare systems are confronted with the challenging task of allocating their scarce resources judiciously and efficiently [4].

In the context of current clinical practice, this challenge has increased physicians’ accountability for pursuing healthcare efficiency, which involves linking outcomes with costs. This evolving role of physicians in promoting healthcare efficiency underscores the growing importance of providing training in healthcare efficiency to future physicians, particularly during their residency training [5–13]. A pivotal aspect of residency training programs is their predominantly clinical, workplace-based approach, where learning mainly occurs through implicit, informal learning [14].

However, within the clinical workplace and literature, various terms denoting healthcare efficiency are nowadays used interchangeably, such as cost-effectiveness, overdiagnosis, overuse, underuse, value-based healthcare, and high-value cost-conscious care [15–21]. The coexistence of these diverse terms and their interpretations regarding healthcare efficiency might suggest that unrelated yet inherently intertwined topics are being addressed. Therefore, we aim to elucidate their meanings, commonalities, and differences, providing a comprehensive understanding of healthcare efficiency to improve medical education. Such knowledge is needed to ultimately discuss the suitability of the different terms in educating (future) physicians to contribute effectively to enhanced healthcare efficiency within their clinical practice. This conceptual understanding should provide a comprehensive perspective on the broad continuum of healthcare efficiency. In medical education, this understanding is important to better prepare (future) physicians to acquire skills needed to balance cost and quality in their decision-making, ultimately contributing to improved healthcare efficiency in clinical practice.

## Methods

Our narrative review involved a critical survey of articles describing and applying healthcare efficiency terminologies. To pursue a high-quality narrative review, the criteria of the Preferred Reporting Items for Systematic Reviews and Meta-Analyses statement were used to report this narrative review wherever possible (see Supplemental Material 1).

### Search strategy

In February 2024, one researcher (LB) searched the PubMed database to retrieve relevant articles for 2019–2024, as we were primarily interested in healthcare efficiency terminologies that are nowadays used in clinical practice. Keywords used in the search strategy reflected healthcare efficiency core elements, using words indicating the gains related to outcomes versus the losses related to resource utilization: (‘outcome’ OR ‘quality’ OR ‘effect’ OR ‘benefit’ OR ‘harm’) AND (‘balance’ OR ‘balancing’ OR ‘consider’ OR ‘considering’ OR ‘analyze’ OR ‘analysis’ OR ‘analyzing’ OR ‘waste’ OR ‘wasting’ OR ‘compare’ OR ‘comparing’ OR ‘achieve’ OR ‘achieving’) AND (‘cost’ OR ‘expenditure’ OR ‘spending’ OR ‘investment’ OR ‘resource’) AND (‘definition’ OR ‘explanation’ OR ‘description’ OR ‘interpretation’ OR ‘specification’ OR ‘concept’ OR ‘idea’ OR ‘principle’).

### Inclusion and exclusion criteria

The articles in this narrative review were included based on the following criteria: articles (1) conceptually focusing on describing and applying a terminology of healthcare efficiency; (2) containing the healthcare efficiency’s core elements of the gains related to outcomes versus the losses related to resource utilization; (3) applying a healthcare efficiency terminology relevant for physicians; and (4) published in the English or Dutch language. Articles with the following characteristics were excluded from the review: empirical studies, practice-oriented articles, and articles specifically targeting a particular population, disease, or context. Such articles were excluded because they primarily focused on testing hypotheses or applying methodologies within specific populations, conditions, or settings. As a result, they often provided limited insight into the conceptual understanding or the terminology used, making them less suitable for contributing to the generic understanding of healthcare efficiency terminology. We refined and validated the criteria by having two authors of this study (WvM and CN) and one research collaborator (Brigitte A.B. Essers, PhD) each independently review a different set of 30 articles. LB also reviewed these articles, after which differences were discussed until consensus was reached.

### Selection and data extraction

**Fig. 1:**
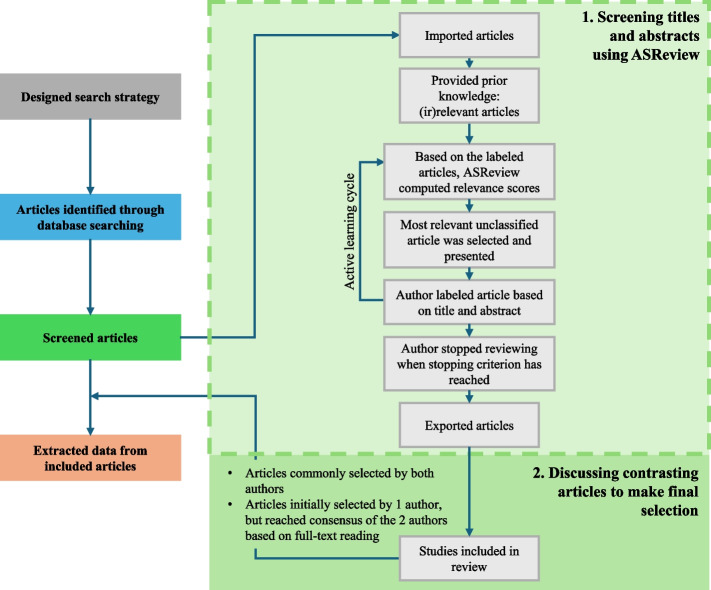
General process related to the methodology and the specific role of ASReview

The article selection process encompassed the following steps: (1) screening titles and abstracts using ASReview, and (2) discussing contrasting articles and reassessing articles that were not commonly selected to make the final selection. Figure 1 shows the general process related to the methodology used in this review and the specific role of ASReview in this process.

#### Screening titles and abstracts using ASReview

We used an open-source artificial intelligence (AI) tool, ASReview (V.1.4) [22]. ASReview employs a machine learning algorithm that prioritizes articles based on their textual proximity to previously identified relevant articles (by the researchers). The tool consequently *reduces the time and effort* required during the initial screening phase but does not *replace* the initial screening of articles by researchers. The use of ASReview involved multiple steps to facilitate the screening phase. Initially, the researchers trained the tool with pre-labeled articles (i.e. prior knowledge). In our review, this prior knowledge included fifteen articles: two relevant articles [23, 24] and thirteen randomly selected irrelevant ones. Subsequently, employing an active learning cycle, ASReview generated a ranking of all unlabeled articles, presenting the most promising article for meeting the inclusion criteria based on the system’s training set generated ‘prior knowledge’. The reviewer then determined the article’s relevance (i.e. relevant or irrelevant) based on its title and abstract, which aligned the eligibility criteria. The tool adapted to this input, reranking all unlabeled articles and suggesting the next highest-ranked article for review. This process continued until the predefined stopping criterion was reached, set on 100 consecutive irrelevant articles. The stopping criterion was left to the individual reviewers, as explained and discussed in a paper addressing the application of ASReview. Although our study included fewer articles to screen, we adhered to the stopping criterion used in that paper [25]. By following this process, it was hypothesized that no further relevant articles remained unseen within the dataset. This screening phase was conducted independently by two authors (LB and SV), who reviewed the titles and abstracts of all identified studies for potential inclusion.

#### Discussing contrasting articles to make final selection

The selections were compared to identify (1) articles commonly selected and (2) articles selected by only one author. Articles agreed upon by both authors were automatically included in the review, while the remaining articles had their inclusions reassessed by consensus of the two authors based on full-text reading. Detailed information regarding the decisions made (labeling the articles as relevant or irrelevant by the two researchers) throughout the screening process of using ASReview can be found in Supplemental Material 2. The final selection of articles consisted of the articles commonly selected by both authors. Plus the articles initially selected by one author, but reached consensus of the two authors based on full-text reading. The data extraction yielded information on general characteristics (i.e. author, domain of medicine, and year of publication) and the terminology relating to healthcare efficiency mentioned in the article.

## Results

**Fig. 2:**
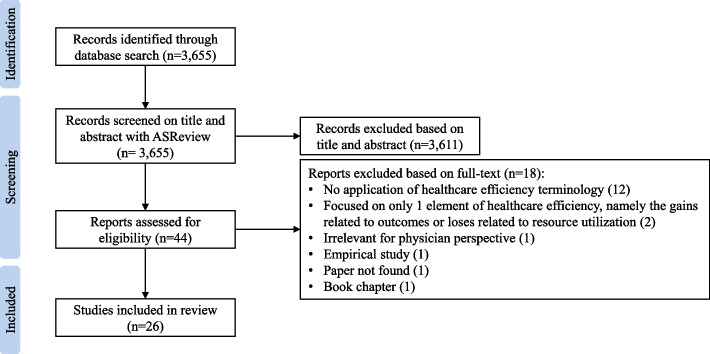
Flowchart of study selection

Out of 3,655 articles identified, 26 studies met the inclusion criteria and were included in our review. The final selection of articles was obtained according to the workflow outlined in Fig. 2. A list of the included articles can be found in Table 1. Our primary focus was on the diverse terms discussed within these articles. Therefore, in the following sections, we delve into these terms and their attributions rather than diving deeper into these articles’ characteristics. First, we describe each term separately. Second, we explain the identified core concept underlying all terms, namely 'value.' Finally, we highlight each term’s distinct perspectives regarding the core’value’ concept.

**Table 1:** Overview of the included studies

Author	Study year	Domain of medicine	Healthcare efficiency term
Bilcke et al. [26]	2021	health economics	cost-effectiveness
Blonda et al. [27]	2021	healthcare policy and health economics	economic evaluation, cost-effectiveness
Castañeda-Orjuela et al. [28]	2020	healthcare policy and health economics	cost-effectiveness
de Jong et al. [29]	2023	clinical medicine and healthcare evaluation	value-based healthcare
de Silva Etges et al. [30]	2023	healthcare policy and management and health economics	value-based healthcare
Dombrádi et al. [31]	2021	healthcare policy and management	value-based healthcare
European Society of Radiology [32]	2019	clinical medicine, healthcare policy, and health economics	value-based healthcare
Garrison Jr et al. [33]	2019	health economics	(QALY-based) cost-effectiveness
Garrison Jr et al. [34]	2019	health economics	value
Howdon et al. [35]	2022	healthcare policy and health economics	economic evaluation, cost-effectiveness
Kazi et al. [36]	2019	healthcare policy and health economics	cost-effectiveness
Kherad et al. [37]	2020	medical education and healthcare policy	overuse (including low-value care, high-value care, overtreatment, overdiagnosis)
Kim et al. [38]	2021	healthcare policy and health economics	low-value care
King J [39].	2021	health economics	economic evaluation, cost–benefit
Maraiki et al. [40]	2022	healthcare policy and health economics	economic evaluation
Miyamoto et al. [41]	2021	health economics	economic evaluation, cost-effectiveness
O’Donnell et al. [42]	2023	healthcare management and policy	value-based healthcare
Pajewska et al. [23]	2020	healthcare management and policy	value-based healthcare
Parikh et al. [43]	2019	healthcare management and health economics	high-value care
Paulden M [44].	2020	health economics	economic evaluation, cost-effectiveness
Pennestri et al. [45]	2019	healthcare management and health economics	value-based healthcare
Pignatti et al. [46]	2021	healthcare policy and health economics	cost-effectiveness
Stanberry et al. [47]	2021	healthcare administration and management	value-based healthcare
Van Muylder et al. [48]	2023	health economics	economic evaluation
Walraven et al. [24]	2021	health economics	value-based healthcare, cost-effectiveness
Zhang et al. [49]	2021	health economics	cost-effectiveness
*QALY *Quality-adjusted life-year			

### Description of healthcare efficiency terminologies

**Table:** 

Based on the included articles, we identified the following four umbrella healthcare efficiency terms (i.e. most commonly used): “cost-effectiveness”, “high-value care”, “low-value care”, and “value-based healthcare”. Some terms overlapped, such as overuse with low-value care and economic evaluation with cost-effectiveness. While other terms were also used in the articles, we focused on those relevant to (future) physicians in clinical practice. This paragraph describes each term separately—cost-effectiveness, high-value care, low-value care, and value-based healthcare—and mentions terms related to the four terms mentioned. Some related terms were not mentioned in the selected articles but were discovered upon further exploration of the included terminologies’ definitions. The umbrella or (most commonly used) terms are in bold, and the related terms are in italics	

#### Cost-effectiveness

*Cost-effectiveness*: [24, 26–28, 33, 35, 36, 41, 44, 46, 49] care that is cost-effective based on an analysis in which costs are related to a single, common effect that may differ between alternative health care programs [15]. The umbrella term is *economic evaluation* [27, 35, 39–41, 44, 48]—a longstanding framework to make the best use of clinical evidence through an organized consideration of the available alternatives’ effects on health, costs, and other effects that are regarded as valuable [15]. Economic evaluations include different types of analysis, i.e. *cost–benefit*, *cost-effectiveness, cost-minimization*, and *cost-utility*. These evaluations can have different outcomes, i.e. monetary outcome for *cost–benefit* and *cost-minimization*, clinical effect outcome for **cost-effectiveness***,* and utility outcome for *cost-utility*). An example of the practical application of *cost-effectiveness* in decision-making is comparing two treatments for aneurysmal subarachnoid hemorrhage: endovascular treatment and neurosurgical clipping.

#### High-value care

*High-value care*: [37, 43] care in which evidence suggests that the probability of benefit exceeds probable harm, or, more broadly, in which the added costs of the intervention provide proportional added benefits relative to alternatives [50]. A related broad term is *right care*—care tailored for optimizing health and wellbeing by delivering what is needed, wanted, clinically effective, affordable, equitable, and responsible in its use of resources [50]. Another related term is *high-value, cost-conscious care, *which aims to assess the benefits, harms, and costs of procedures to deliver care that adds value to the patient [6]. As evident by its nomenclature, it is grounded on *high-value care*. It also indicates physicians’ responsibility to practice cost-consciously [5]. *High-value, cost-conscious care *is mainly described in research calls to train (future) physicians in the competency of understanding their responsibility for practicing cost-consciously and the need for stewardship of resources [5, 6, 8, 21]. An example of the practical application of *high-value care* in decision-making is prescribing a generic statin for high cholesterol instead of a branded statin variant.

#### Low-value care

*Low-value care*: [37, 38] care in which evidence suggests it adds no or very little benefit for patients, or the risk of harm exceeds the probable benefit, or, more broadly, the added costs of an intervention do not provide proportional added benefits [50]. Related terms are *misuse*—when an appropriate service has been inappropriately applied and a preventable complication occurs, so the patient consequently does not experience the full potential benefit of the service [51]; *overdiagnosis/overtesting* [37]*—*diagnosing abnormalities or symptoms that are indolent, non-progressive, or regressive that will not cause considerable distress or early death [18]. *Overdiagnosis/overtesting* often leads to unnecessary interventions; *overtreatment* [37]—delivering particular treatment types of inappropriate procedures [17]. *Overtreatment* leads to excessive or unnecessary interventions*; overuse* [37]—when a service is provided under circumstances in which its potential for harm exceeds the possible benefit [51]. *Overuse* encompasses a broader concept, including diagnostic tests, procedures and treatments that provide no net benefit, and *underuse*—failing to provide a healthcare procedure that is highly likely to generate a favorable outcome for a patient [19, 51]. An example of the practical application of *low-value care* in decision-making is performing routine imaging scans for low-back pain in all patients, even in those without red-flag symptoms.

#### Value-based healthcare

*Value-based healthcare*: [23, 24, 29–32, 42, 45, 47] care that aims to achieve high value for patients, with value defined as the patient-relevant health outcomes (health gains) achieved per unit of cost spent for the entire care cycle [16, 52–54]. Related terms such as *value-based procurement* are derived from the *value-based healthcare* term. An example of the practical application of *value-based healthcare* in decision-making is opting for palliative care over aggressive interventions for a patient with advanced heart failure after discussing what is considered valuable from the perspective of this individual patient.

For the readability of this paper, we use the four umbrella (most commonly used) terms over the related one(s) described above in the remainder of the text.

### Core concept of ‘value’ underlying healthcare efficiency terms

**Table:** 

In situations of scarcity concerning healthcare efficiency, selecting procedures solely based on their effectiveness or costs is futile. Performing procedures based on effectiveness without considering their costs can lead to unsustainable healthcare expenditures. Conversely, selecting procedures based on cost(s) (reduction) without considering their effectiveness can result in lower quality of care and, ultimately, higher health expenditures in the long run. Therefore, the concept of ‘value’ is central to all healthcare efficiency terminologies and is often described as a trade-off between benefits in health outcomes against resource use in healthcare costs. This paragraph elaborates on how value is described across the terms	

The term *cost-effectiveness* refers to a method or tool that formally assesses the incremental value of care by analyzing costs to the effectiveness of different alternatives [15, 24]. As such, *cost-effectiveness* primarily informs resource allocation decisions on a population-level. The term *value-based healthcare* concentrates on achieving high value for patients as the principal goal for stakeholders in healthcare—creating more value for money [16, 54]. The value of care in this term is described as patient-relevant health outcomes (quality of care) achieved per unit of cost spent for the entire care cycle (cost of care) [16, 52–54].

Healthcare procedures can be broadly grouped into two value-related categories: *high***-** and *low-value care*. The term *high-value care *focuses on providing procedures of high value, which is defined as care in which evidence suggests the added costs of the intervention provide proportional added benefits relative to alternatives [50]. In short, *high-value care* adds value and should be embraced and scaled up. Conversely, the term *low-value care *focuses on eliminating procedures of low value, which is defined as care in which evidence suggests the added costs of the intervention do not provide proportional added benefits [50]. Simply put, *low-value care* provides minimal or no value and should be eliminated. *Low-value care* wastes limited resources. In order to maximize value, it is essential to find the right balance between continuing and investing in areas where high patient value (*high-value care*) can be achieved and discontinuing and disinvesting in areas where it cannot (*low-value care*).

### Terminologies’ distinct perspectives regarding the concept of ‘value’

**Table:** 

Value is central in healthcare efficiency terminologies, but their meaning attributed to what constitutes value are distinct. On the one hand, decision-making can be guided by allocating scarce resources in accordance with the entire population’s needs—maximizing value at the population-level. On the other hand, decision-making can be guided by patient-centricity that represents the patient’s needs—maximizing value at the individual patient-level. This paragraph describes the two distinct perspectives regarding the core concept of value	

#### Maximizing value at the population-level

Within *cost-effectiveness*, the value of a procedure or intervention is expressed as a ratio, i.e. incremental cost-effectiveness ratio (ICER), which is the difference in cost divided by the difference in outcome [15]. This is often described as quality-adjusted life-years (QALYs). A QALY combines health-related quality of life, based on the Euroqol-5D, with survival [55]. The utility scores for the Euroqol-5D are obtained by asking a society sample to value health states, thus valuing health from a societal perspective. This perspective includes not only costs of the procedure itself (i.e. direct costs) but also downstream costs resulting from the procedure (i.e. indirect costs, such as productivity loss and informal care costs) [56]. The incremental cost per QALY is interpreted in perspective of a threshold, representing the amount of money society is willing to pay for an additional QALY, to determine whether an intervention is cost-effective compared to its alternative.

**Fig. 3:**
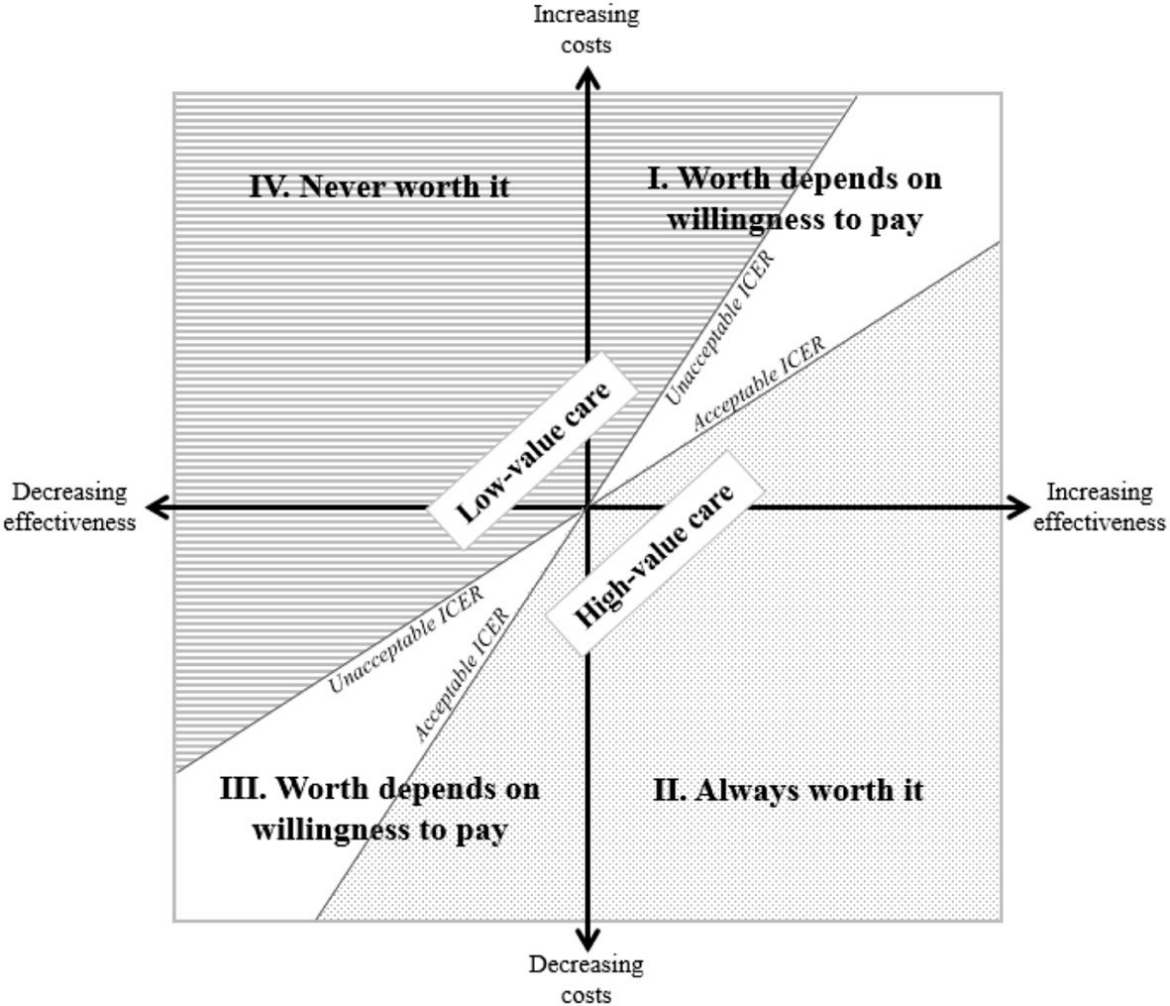
The cost-effectiveness plane with its four quadrants based on Sacristán [59], with modifications. ICER = incremental cost-effectiveness ratio

In this regard, resource allocation decisions can be made on a population-level to maximize value in situations of scarcity and finite budgets using the cost-effectiveness plane (see Fig. 3). A cost-effectiveness plane is a four-quadrant visualization representing differences in costs and effects between alternative procedures. Quadrant II represents *high-value care* (more effective, fewer costs), and quadrant IV represents *low-value care* (less effective, more costs) respectively, while quadrants I and III require further consideration based on the described threshold. Using such cost-effectiveness evidence, one can eliminate *low-value care*: ineffective care that is less effective and more expensive than alternatives, or not implementing care with an unacceptable incremental (or decremental cost-effectiveness) threshold. However, there is a more ambiguous ‘grey area’ in-between *high-value* and *low-value care*: care that offers little benefit to most patients, care for which the balance between benefits and harms varies substantially among patients, and care with insufficient or lacking evidence to assist in decide which patients, if any, might benefit and by how much [17]. Supplemental Material 3 provides detailed information regarding cost-effectiveness analysis.

#### Maximizing value at the individual patient-level

*Value-based healthcare *focuses on organizing and improving care at the patient-level [16]. It is described as a holistic, patient-centered understanding of value concentrating mainly on patient-physician interaction [24]. Value is herein equated as individual patient-relevant health outcomes in relation to the total costs of attaining those outcomes [16, 54]. However, the trade-off between outcomes and costs is not per se expressed in a single number or ratio. *Value-based healthcare *encompasses primarily personal value and goal-oriented care, meaning that (future) physicians should consider ‘what matters most’ for individual patients when making decisions [16, 52, 56]. Patient-relevant outcomes are the notion’s numerator and can be a multidimensional condition-specific construct—for medical conditions, no single outcome may capture the results of care [16].

Similar to cost-effectiveness, *value-based healthcare* inherently means preserving the delivery of care that provides good value by eliminating sources of waste (*low-value care*) and delivering care that provides benefits commensurate with costs (*high-value care)*. Verkerk and colleagues [57] described that it is important to understand the individual patient’s needs and preferences for (future) physicians’ decision-making in the context of scarce resources and contemporary healthcare efficiency. Whether care is of low or high value is thus determined on an individual level by the healthcare professional and the patient based on the best available cost-effective evidence and the patient’s preferences [58]. Table 2 briefly summarizes the healthcare efficiency terms and the explained value perspectives.

**Table 2:** Summary of the healthcare efficiency terms and ‘value’ perspectives

**• Cost-effectiveness** strives to maximize value at the population-level; value is defined from a societal perspective to efficiently allocate resources to societal needs.
**• Value-based healthcare** strives to maximize value at the individual patient-level; value is defined from a patient perspective to address individual patient needs and preferences.
**• Cost-effectiveness** and **value-based healthcare** aim to respectively deliver care that adds value (**high-value care) **and avoid care that adds no value** (low-value care)**.
• To determine whether care can be considered as low or high value, value is based solely on cost-effectiveness results or considers patient preferences as well.

## Discussion

To identify which healthcare efficiency term is most suitable for medical education in equipping physicians to contribute to healthcare efficiency delivery in clinical practice, this narrative review aimed to provide an overview and understanding of these terminologies, highlighting their meanings, commonalities, and differences. To the best of our knowledge, this is the first paper to offer guidance regarding gaining a better understanding of healthcare efficiency for (future) practicing physicians.

### Terminologies’ suitability for healthcare efficiency in clinical practice

Concerning decision-making in the clinical workplace for (future) physicians, an obstacle of cost-effectiveness results, such as the incremental costs per QALY, might be that this information may be too difficult to use in their interactions with individual patients. In addition, it is questionable whether all relevant health domains are captured within the QALY, which is based on a generic health-related quality of life questionnaire. Moreover, the absence of formally defined thresholds for decremental cost-effectiveness compounds the challenge of applying this framework in clinical practice [59]. However, such thresholds are important at the population-level in resource-limited settings as the saved resources allow reallocation of resources to higher-value alternatives. In contrast, value-based healthcare may be accessible and attractive to (future) physicians due to its focus on measuring patient-relevant outcomes, thereby potentially reducing waste. However, the challenge within value-based healthcare may be its abstractness; they generally cannot exactly establish care’s value related to both costs and outcomes because of the personalized nature [60]. It may thus insufficiently facilitate (future) physicians to deal with scarce resources in the clinical workplace at the population-level. The road to patient-physician interactions might be challenging and ambitious when applying the term and concept of cost-effectiveness. In contrast, with value-based healthcare, doubts may arise about whether it can sufficiently contribute to solidarity, affordability, and sustainable healthcare in practice.

While cost-effectiveness and value-based healthcare operate at different levels, they are not mutually exclusive. An integrated approach, in which considering cost-effectiveness principles inform broader resource allocation, while the perspective of value-based healthcare ensures patient-centered decision-making, may help bridge the gap between economic considerations on the one hand and clinical practice on the other. For example, guidelines based on cost-effectiveness and subsequently translated into a decision aid, can help (future) physicians provide care in clinical practice.

#### Balancing societal and individual patient perspectives

Ideally, the societal perspective on value on the one hand and the individual patient’s perspective on value on the other hand are in the same direction. In practice, however, tension or even conflict between personal patient values and allocative societal values might emerge [61, 62]. Still, a clear understanding of cost-effectiveness results is relevant to patients and (future) physicians within the clinical workplace to choose a procedure with value for money. In doing so, it should be recognized that the costs of care (e.g. diagnostic or treatment costs) are often hidden, unknown, or poorly understood by patients or even (future) physicians [63]. A shared decision-making process between patients and (future) physicians may facilitate decision-making about cost-effectiveness information on a case-by-case basis. Hence, it is essential for (future) physicians to understand healthcare efficiency’s meaning, how to make trade-offs between outcomes and costs, and, subsequently, how to communicate this with patients appropriately.

#### Influence of contextual factors

Furthermore, although the different healthcare efficiency terms describe which care can be considered value for money, the influence of contextual factors on adopting efficiency-driven practices must be acknowledged. Multiple studies identified that factors such as payment systems, organizational characteristics, fear of malpractice litigation, state of scientific knowledge, and a “more is better” culture affect low-value care [64–66]. Besides, Bock and colleagues [67] showed that multiple contextual factors influence residents’ decision-making concerning low-value care over a wide range from the individual resident (e.g. compulsion to act), to interpersonal (e.g. patients’ expectations), to organizational (e.g. standards of practice), and to the environmental and socio-political system (e.g. societal sentiment). While educating (future) physicians on the principles of healthcare efficiency seems important, addressing these contextual factors is likewise critical to ensure healthcare efficiency delivery in clinical practice.

### Implications for practice and medical education

In contemporary healthcare, (future) physicians are advocates of individual patients on the one hand, but they are also stewards of finite resources on the other. By following the principles of cost-effectiveness, limited resources are allocated to the population’s needs by continuing to invest in high-value care and disinvesting in low-value care. Value-based healthcare’s vital and supplementary role ensures an important nuance in creating individualized care plans representing patients’ needs. Therefore, we advocate that medical education should stimulate and facilitate (future) physicians to acquire sufficient knowledge of the different terminologies of healthcare efficiency, and obtain an understanding of diagnostic and treatment procedures’ costs in relation to contemporary evidence-based guidelines. Additionally, (future) physicians should be trained to acquire the adaptive expertise of applying individually tailored professional communication skills in the process of shared decision-making to practice healthcare efficiency in the clinical workplace. 

#### Understanding healthcare efficiency terms

Medical education—via formal and informal learning—needs to instil healthcare efficiency’s basics to (future) physicians, including the different terminologies. Since practice behaviors developed during residency training often persist throughout a career [5], we encourage, in particular, to prepare future physicians in residency training programs to assess and concentrate on the value of care (benefits that are commensurate with their costs—‘when is it worth it’) instead of only the benefits or only the costs. Medical education might focus on teaching future physicians about healthcare costs considering evidence-based guidelines. This will enable them to integrate cost-effectiveness information into treatment decisions, ensuring the delivery of high-value care to patients in general. For example, introducing and applying the cost-effectiveness plane by analyzing case studies in the formal curriculum of residency programs can help future physicians understand the principles of the different terminologies. Likewise, using a flipped classroom approach allows residents to review the cost-effectiveness plane in advance, enabling active discussions and problem-solving during case-based sessions. In addition, supervisors can facilitate discussions of real patient cases and compare different treatment options based on their costs and benefits. Such interactive reflective practice helps train future physicians in determining which offers the best value for the individual patient and the healthcare system.

#### Enhancing communication skills

Medical education should give (future) physicians the opportunity to develop relevant skills that support a cost-informed, evidence-based, shared decision-making process with individual patients. Herein, the concept of adaptive expertise seems relevant. Adaptative expertise involves equipping (future) physicians with the ability to flexibly apply knowledge and skills to effectively solve varied and new situations [68]. This adaptive approach ensures that (future) physicians can engage in informed, personalized, shared decision-making that prioritizes both clinical effectiveness and cost considerations. In doing so, for example, it may be helpful to jointly, patients and physicians, negotiate and determine the individual patient’s preferred communication style for discussing the cost-effectiveness of treatment options or conveying the message of why a treatment or diagnostic test is sometimes of low value and even harmful.

After all, being a (future) physician is not just about determining patients’ benefit; it is also about understanding value from a broader societal perspective. An evidence-based, shared decision-making process informed by high-quality information about the risks, benefits, and costs of care is such a value-driven discussion. Hence, we hope our overview of healthcare efficiency’s terminologies enables (future) physicians to balance societal costs and individual patients’ outcomes in healthcare and communicate this process to patients.

### Recommendations for future research

We recommend conducting additional qualitative research to examine how various terms influence physicians’ attitudes and behaviours in decision-making processes to validate our findings. Presenting alternative interpretations to physicians and discussing their reactions in semi-structured focus groups could be beneficial in identifying which terminology most effectively promotes attitudes that lead to the desired outcome of healthcare efficiency. Herein, focus group discussion based on vignettes could be useful, allowing varying emphasis on one of the terminologies to be discussed. Furthermore, although the different healthcare efficiency terms describe which care can be considered value for money, there may be contextual reasons to provide procedures that might be less effective and potentially also not cost-effective in clinical practice. Many contextual factors contribute to low-value care delivery, such as patients’ preferences or pressure, extrinsic financial pressures, or lack of care providers’ time [2, 65]. Concerning healthcare efficiency, the facilitators and barriers that either stimulate or hamper value-driven decisions need to be tackled. Therefore, these contextual factors need to be further explored, focusing on factors which most significantly drive low-value care. Examining the latter factors from the perspective of both the (future) physicians and patients seems valuable by preferably participant observation and subsequently individual semi-structured interviews, as research shows that patients influence residents’ decision-making regarding low-value care [67]. In addition, our paper primarily focused on conceptual and theoretical literature to allow a comprehensive synthesis of key terminologies relevant to medical education. Future research could conduct a systematic review of empirical and practice-oriented studies to offer a practical perspective on how healthcare efficiency terms are utilized and interpreted in different settings.

### Affordances and limitations

This paper provides an unprecedented overview of healthcare efficiency terminologies that can guide both medical education and clinical practice, using an innovative tool to aid article selection. However, this paper should also be considered in the light of its limitations. First, the use of the AI tool introduces some level of uncertainty. Some relevant articles may have been missed due to the use of the stopping criterion. Indeed, once this point was reached during screening, the remaining articles were no longer reviewed, making this approach sensitive to possible mistakes when only one researcher screens articles. In addition, the use of the machine learning algorithm can introduce biases during the process of prioritizing studies. Such bias may cause certain types of studies to be over- or under-prioritized, affecting the researcher's outcomes. To address these issues and to improve validity and reliability, the algorithm was trained on multiple prior knowledge articles, and two authors independently assessed the relevance of the articles to ensure complete double screening. To minimize the primary risk of missing relevant articles when using an AI-tool while screening the articles, future research could make use of a recently published paper that presents a procedure that combines an eclectic mix of stopping heuristics [69].

Second, selection bias of the described terms cannot be excluded due to the variability of healthcare efficiency terminologies and the evolving healthcare field. Although thus prone to subjectivity, we strived to counteract the selection bias through extensive discussions related to the formulation of the in- and exclusion criteria within the research team consisting of diverse backgrounds to minimize this risk and to conform to existing important literature.

Third, we conducted a narrative rather than a systematic review, meaning it is not all-inclusive. Plus, healthcare efficiency is extremely broad, and well-known healthcare efficiency initiatives, such as “Choosing Wisely”, are nowadays commonly mentioned and adopted within the clinical practice [70]. However, after careful discussion within the research team, we have decided not to include those in the article since these initiatives use the terms we describe in this paper. Conclusively, we have focused on identifying the current debate's most significant and overarching terms.

## Conclusions

This narrative review provides an overview and understanding of different healthcare efficiency terminologies (i.e. cost-effectiveness, high-value care, low-value care, and value-based healthcare) and discusses their suitability for medical education in equipping (future) physicians to enhance decision-making. The concept of ‘value’ is central within all healthcare efficiency terminologies. Still, they differ mainly in their perspectives on what constitutes value in decision-making: maximizing value at the population- or individual patient-level. Applying the terminologies in clinical practice, however, pose challenges. Cost-effectiveness might be too rigid for patient-physician interactions, while value-based healthcare may equip physicians insufficiently to contribute to sustainable healthcare due to its oversimplification of the costs and outcomes of care. Both terms aim to deliver care that adds value (high-value care) and avoid care that adds no value (low-value care). In contemporary healthcare, (future) physicians are both stewards of finite resources and advocates of individual patients. Hence, we suggest that medical education teaches and trains them to integrate the principles of the healthcare efficiency terminologies by introducing the cost-effectiveness plane to analyze case studies. Additionally, case-based discussions can help determine which treatment option offers the best value for the individual patient and the healthcare system. Furthermore, we suggest that medical education should facilitate and stimulate (future) physicians to acquire (more in-depth) knowledge on diagnostic and treatment procedures’ costs in the context of contemporary evidence-based guidelines. Medical education should also focus on improving communication skills supporting a healthcare efficiency-driven open shared decision-making adaptively tailored to individual patients. Future research may explore how the various terminologies influence physicians’ attitudes and behaviours in decision-making. Additionally, contextual factors—such as patient preferences, financial pressures, and time constraints—that contribute to low-value care can be further examined from both physicians' and patients' perspectives.

## Supplementary Information

Supplementary Material 1.

Supplementary Material 2.

Supplementary Material 3.

## Data Availability

No datasets were generated or analysed during the current study.

## Abbreviations

AI: Artificial Intelligence
QALY: Quality-Adjusted Life-Years
ICER: Incremental Cost-Effectiveness Ratio
