# Supplementary material for: Understanding healthcare efficiency—an AI-supported narrative review of diverse terminologies used
Source: BMC Med Educ. 2025 Mar 20;25:408. doi: 10.1186/s12909-025-06983-5 (PMC11924740; doi:10.1186/s12909-025-06983-5)
Supplement: Supplementary file 3 — Supplementary Material 3. [file 12909_2025_6983_MOESM3_ESM.docx]

**Supplemental Material 3.**

**The cost-effectiveness plane**

A cost-effectiveness plane (see Figure 2 in main text) is a four-quadrant visualization that represents differences in costs and effects between alternative procedures. Quadrant I reflects procedures that are more effective and have more costs; quadrant II procedures that are more effective and have fewer costs; quadrant III procedures that are less effective and have fewer costs; and quadrant VI procedures that are less effective and have more costs. Procedures within quadrant IV are considered low-value care and should be eliminated. In contrast, procedures within quadrant II are considered high-value care and should be provided. However, decisions regarding quadrants I and III are less evident. In quadrant I’s cases, the healthcare system’s willingness to pay threshold should be established concerning an increased benefit (acceptable ICER). In the United States, the Institute for Clinical and Economic Review defined a threshold of $100,000 and $150,000 per QALY gained.^1^ Furthermore, England’s’ National Institute for Health and Care Excellence proposed the ICER’s lower limit of £20,000 and the upper limit of £30,000 per QALY.^1^ Procedures below the lower limit are likely to be accepted, while procedures above the upper limit are usually rejected. In quadrant III’s cases, procedures whose effectiveness is slightly lower but significantly have fewer costs compared to their alternative should be accepted (acceptable decremental CER).^2^ These procedures have the potential to add value to the system as the resources saved permitting resource reallocation towards higher-value alternatives. Nevertheless, decremental cost-effectiveness thresholds have not been defined formally.^3^ In conclusion, the cost-effectiveness plane strives to provide robust evidence to inform value-driven choices. It ensures that the benefits of implemented and delivered procedures exceed their opportunity costs to allocate resources to finance procedures that generate high value for patients eventually.

**References**

1. Thokala P, Carlson JJ, Drummond M. HTA’d in the USA: A Comparison of ICER in the United States with NICE in England and Wales. J Manag Care Spec Pharm JMCP. 2020;26:1162–70.
2. Nelson A, Cohen J, Greenberg D, Kent D. Much cheaper, almost as good: decrementally cost-effective medical innovation. Ann Intern Med. 2009;151:662.
3. Sacristán JA. How to assess the value of low-value care. BMC Health Serv Res. 2020;20:1000.
